# The Effect of Severe Varus Deformity on Clinical and Radiographic Outcomes in Mechanical Aligned Total Knee Arthroplasty with Medial Stabilizing Technique

**DOI:** 10.3390/jcm13061595

**Published:** 2024-03-11

**Authors:** Sung-Sahn Lee, Jewon Jung, Hanbit Kim, Jinwoo Kim, In Geol Jung, Jiin Kim, Young-Wan Moon

**Affiliations:** 1 Department of Orthopaedic Surgery, Ilsan Paik Hospital, School of Medicine, Inje University, Goyangsi 10380, Gyeonggido, Republic of Korea; sungsahnlee@gmail.com; 2 Department of Orthopaedic Surgery, Samsung Medical Center, School of Medicine, Sungkyunkwan University, Seoul 06351, Republic of Korea; jewon88.jung@samsung.com (J.J.); hanchovit.kim@samsung.com (H.K.); jinwoo87.kim@samsung.com (J.K.); ig.jung@samsung.com (I.G.J.); zeein.kim@samsung.com (J.K.)

**Keywords:** patellar fracture, tension band wiring, patellar height, risk factor

## Abstract

**Background:** The purpose was to compare the clinical and radiographic outcomes between preoperative mild and severe varus deformity after total knee arthroplasty (TKA) with medial stabilizing technique (MST). **Methods:** We retrospectively analyzed 158 knees of 125 female patients with a 2-year follow-up who underwent mechanically aligned TKA with MST between April 2018 and February 2021. Patients were divided into two groups; the severe varus group was defined as one with preoperative hip-knee ankle (HKA) angle ≥ 15° and the mild varus group with HKA angle < 15°. Pre- and post-operative clinical outcomes (Western Ontario and McMaster University Osteoarthritis Index, Knee Society Knee Score) and radiographic outcomes (medial proximal tibial angle (MPTA), HKA angle, lateral distal femoral angle (LDFA), joint line distance, and femoral component rotation angle) were compared between the groups. **Results:** Among the 158 knees analyzed, 131 and 27 were allocated to the mild and severe varus groups, respectively. Preoperative data showed that the MPTA (84.7° ± 2.8° vs. 80.7° ± 3.2°, *p* < 0.001) was significantly less in the severe varus group. In postoperative data, clinical outcomes were not different between the groups. Joint line distance (18.4 mm ± 2.8 mm vs. 18.6 mm ± 2.7 mm, *p* = 0.676) was also not significantly different. Femoral component rotation angle (−1.7° ± 1.0° vs. −1.0° ± 1.3°, *p* = 0.018) was more externally rotated in the severe varus group. **Conclusions:** Severe varus group showed comparable clinical and radiographic outcomes to that of mild varus group after mechanically aligned TKA with MST.

## 1. Introduction

Traditionally, obtaining a rectangular gap balance is critical to successful mechanically aligned (MA) total knee arthroplasty (TKA) [1]. Medial or lateral soft tissue release is frequently required to achieve rectangular soft tissue balance [2]. Medial structural release is required in knee osteoarthritis with varus alignment. Proper osteophyte resection and the soft tissue release including posteromedial joint capsule and deep medial collateral ligament (MCL) are enough to gain rectangular shaped mediolateral gap balance in mild varus knee deformities. However, extensive medial soft-tissue release is required in severe varus knee deformities [3]. Adverse effects can occur after an extensive medial release during TKA. Excessive MCL release is associated with midflexion instability and joint line elevation, which are correlated with postoperative knee pain or poor functional outcomes [4]. Therefore, TKA using the medial stabilizing technique (MST) was introduced to minimize the medial side release and accept residual laxity of lateral side. TKA with MST showed good postoperative outcomes, without joint instability or line elevation have been reported [5,6].

TKA for severe pre-operative varus deformities can be challenging [7]. Postoperative outcomes after MA TKA could be poor in patients with severe preoperative varus deformity because restoration of neutral alignment might be more challenging [8,9]. Some studies have reported that clinical outcomes are not significantly lower in patients with severe knee varus than in those with mild varus deformities after MA TKA [8,10]. Although numerous studies have reported the outcomes after TKA with MST, few have investigated the effect of severe varus alignment on postoperative patient outcomes after MA TKA with MST.

Therefore, the purpose of this study was to compare the clinical and radiographic outcomes between mild and severe preoperative varus deformities after MA TKA with MST. We hypothesized that patients with severe varus deformity would show clinical and radiographic outcomes comparable to those with mild varus alignment.

## 2. Materials and Methods

### 2.1. Study Design and Patients

This retrospective comparative study used prospectively collected data. Patients who underwent mechanically aligned TKA with MST between April 2018 and February 2021 were evaluated. The patients performed surgery using posterior-stabilized (PS) Physica prosthesis (Lima Corp., Udine, Italy) was enrolled. The extension gap-first gap technique was performed and all TKAs targeted mechanical alignment.

The inclusion criteria were (1) osteoarthritis of medial compartment rather than inflammatory arthritis, including rheumatoid arthritis; (2) preoperative varus knee (preoperative hip-knee-ankle (HKA) angle > 0°), and (3) follow-up more than 2 years. The exclusion criteria were (1) extra-articular deformity due to previous injury of the femur or tibia and (2) inadequate clinical or radiographic data. The patients who enrolled the current study were divided into ‘mild varus group’ (<15°) and ‘severe varus group’ (≥15°) depended on preoperative hip-knee-ankle (HKA) angle as the definition of previous studies. The protocol of the study was approved by the Institutional Review Board (SMC 2020-06-158, Samsung Medical Center, Seoul, Republic of Korea) and written informed consent was gained.

### 2.2. Surgical Technique

All TKAs were done by the senior author (Y.-W.M.). A pneumatic tourniquet was routinely applied from the incision to the wound closure. After skin incision, a medial parapatellar approach was used for arthrotomy. Meticulous removal of the osteophytes was performed. The deep MCL was released to create a space for implantation. Other medial soft tissue releases were not performed and lateral physiological laxity was allowed to avoid excessive medial release, as in previous studies [5,11]. Distal femoral and proximal tibial cuttings were performed in a plane perpendicular to the mechanical axes of the femur and tibia, respectively. The target tibial slope was 3°. Anterior and posterior femoral cuts were made in a plane parallel to the proximal tibia. Surgical transepicondylar axis (sTEA) was routinely marked during surgery. To avoid an excessively externally rotated femoral component position, the upper limit of the femoral component external rotation was set within three degrees of the sTEA. After bone cutting, a posterior-stabilized (PS) Physica prosthesis (Lima Corp., Udine, Italy) was implanted. Patellar resurfacing was not observed.

One day after the TKA, isometric quadriceps, active ankle, and straight leg-raising exercises were initiated. Knee flexion exercise were started 2 days after surgery from 0° to 120°. None of the patients required manipulation under anesthesia.

### 2.3. Clinical and Radiographic Assessments

The range of motion (ROM) was obtained using a goniometer. The preoperative and 2-year postoperative Western Ontario and McMaster Universities Osteoarthritis (WOMAC) index, the Knee Society Knee Score (KSKS), and the Knee Society Function Score (KSFS) were collected and analyzed [12,13,14,15]. Preoperative and postoperative outcomes were compared. KSKS, KSFS, and WOMAC index were compared between the groups. Revision surgeries for any reason were investigated during total follow up period. 

**Figure 1:**
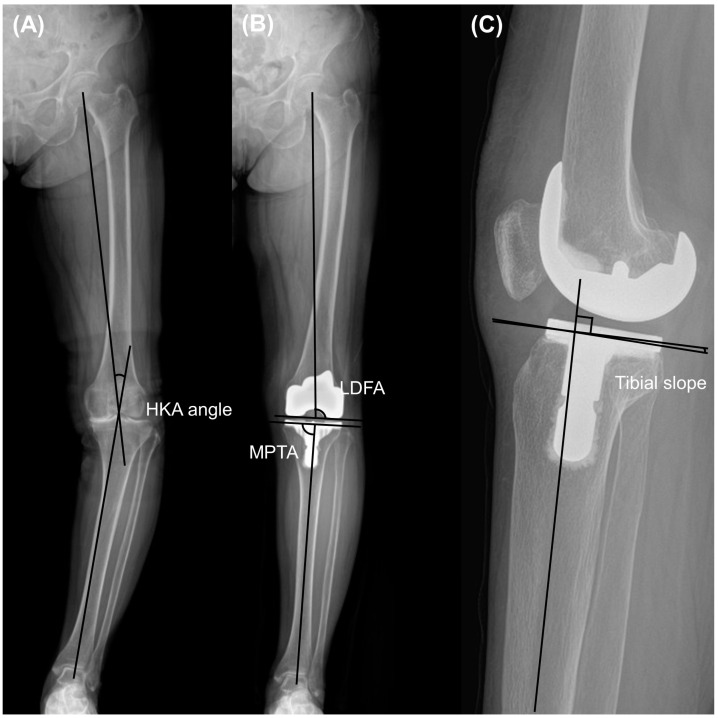
Measurement included (**A**) hip-knee ankle (HKA) angle, (**B**) lateral distal femoral angle (LDFA) and medial proximal tibial angle (MPTA) on whole-leg standing radiographs, and (**C**) tibial slope on lateral view radiographs.

The radiographs obtained before and two years after the surgery were analyzed. The analysis involved measuring the Hip-Knee-Ankle (HKA) angle, which is the angle formed between the line connecting the center of the femoral head to the center of the knee and the line from the center of the knee to the center of the talus, using whole-leg standing radiographs. Varus and valgus were designated as positive and negative HKA angles, respectively [16]. Additionally, the lateral distal femoral angle (LDFA) and medial proximal tibial angle (MPTA) were assessed to examine the preoperative phenotype and implant position in the coronal plane [17,18]. The LDFA was defined as the angle between the line parallel to the femoral condyle and the mechanical axis of the femur. The MPTA was defined as the angle between the line parallel to the tibial plateau or component and the anatomical axis of the tibia (Figure 1). Knee preoperative phenotypes were classified as previous investigation [19]. Alignment variation was defined as a 3° range for any of these angles. The mean values of these phenotypes represented 3° increments in the angle, starting from the overall mean value observed in a young non-osteoarthritic population (NEU_HKA_0° = 0°, NEU_LDFA_0° = 87°, NEU_MPTA_0° = 87°). The nomenclature of the phenotypes was constructed as follows: the first part (NEU, VAR, and VAL) indicated the alignment direction; the second subscripted part (HKA, LDFA, and MPTA) represented the measured angle, and the last part (0°, 3°, 6°, etc.) indicated the mean deviation of the phenotype from the mean value. Postoperative HKA angle outliers were defined as greater than 3° or less than −3°, and LDFA or MPTA outliers were defined as greater than 93° or less than 87°, as described in a previous study [20]. The distribution rates of the HKA, LDFA, and MPTA phenotypes were compared between the two groups. Correlation analysis was performed for the preoperative HKA angle and other pre- and postoperative variables, including LDFA, MPTA, tibial slope, femoral condylar offset, joint line distance, and femoral component rotation angle. The tibial slope was quantified as the angle formed between the mid-diaphysis line of the tibia and the line representing the posterior inclination of the tibial plateau or implant in the lateral view.

**Figure 2:**
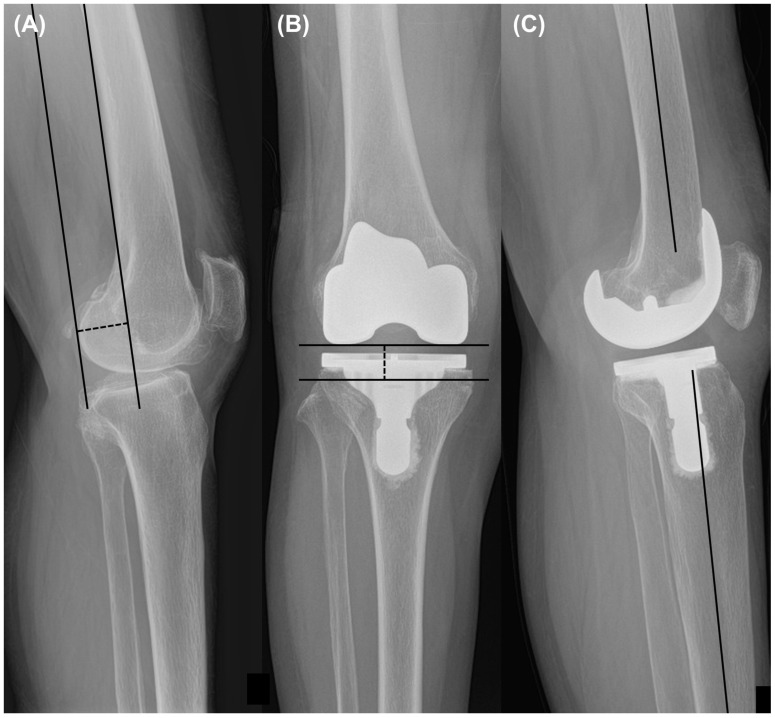
Measurement of (**A**) femoral posterior condylar offset, (**B**) joint line distance on anteroposterior radiographs, and (**C**) extension angle on full extension lateral view radiographs.

The femoral posterior condylar offset is measured from the posterior condyle to the posterior cortical margin as the distance (Figure 2) [21]. The extension angle on a radiograph was highly reproducible and demonstrated greater accuracy than the measurement on a goniometer [22]. This angle was measured as the angle between the distal femoral axis and the proximal tibial diaphyseal axis, that is, the line connecting the midpoints of the outer cortical diameter at 5 cm and 15 cm proximal or distal to the joint line on the full extension lateral view. It was also measured pre-and postoperatively [21]. A positive value denoted flexion contracture, while a negative value signified hyper-extension. Joint line distances were measured preoperatively and postoperatively as the distance from the apex of the fibular head to the tibial plateau or femoral component, respectively [23]. 

**Figure 3:**
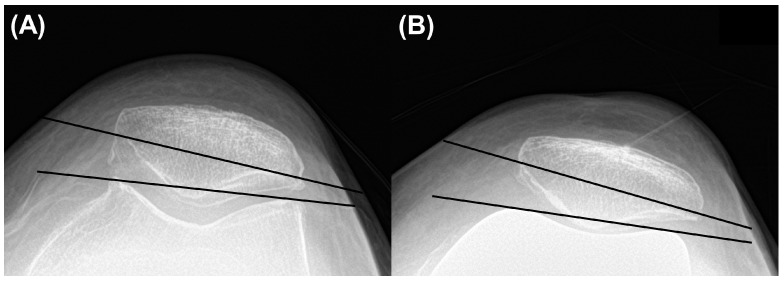
Measurement of (**A**) preoperative and (**B**) postoperative patellar tilt angle.

The patellar tilt angle was measured the angle between the line drawing the anterior limits of the femoral components or condyles and the line from one corner of the patella to the other on knee Merchant view. A positive value meant lateral translation of patella [24] (Figure 3).

Postoperative computed tomography (CT) scan was routinely performed to analyze the rotational alignment of the femoral component. The femoral component rotation angle was defined as the angle between clinical transepicondylar axis (cTEA) and the line connecting the most prominent points of the medial and lateral femoral posterior condyles. The greater measurement value indicated a more externally rotated femoral component, whereas a lower value indicated an internally rotated femoral component. The radiographic measurements were analyzed employing a picture archiving and communication system (Centricity; General Electric, Chicago, IL, USA). Two independent orthopedic surgeons conducted the assessment to ascertain inter-observer reliability. To ensure intra-observer reliability, two observers repeated the measurements at 6-week intervals. Intraclass correlation coefficients (ICCs) were utilized to evaluate the inter- and intra-observer reliabilities for alignment measurements. All inter- and intra-observer ICCs demonstrated a high level of agreement (>0.80). In congruence with the clinical evaluations, a comparative analysis was performed on preoperative, postoperative, and radiographic outcomes between the groups. Furthermore, preoperative and postoperative outcomes were also compared.

### 2.4. Statistical Analysis

The Shapiro-Wilk test was used to evaluate the normality of the distribution. Pearson’s correlation analysis was performed to investigate the correlation between the preoperative HKA angle and other variables, including the LDFA, MPTA, tibial slope, femoral condylar offset, joint line distance, and femoral component rotation angle and to identify the relationship between the postoperative patellar tracking (patellar tilt angle) and postoperative WOMAC, KSKS, and KSFS. The chi-square test was used to compare the distributions of the HKA angle, LDFA, and MPTA phenotypes between the groups. Paired *t*-tests for continuous variables and chi-square tests for categorical variables were used to compare preoperative and postoperative outcomes. Student’s t-test or chi-squared test was used to compare demographic data and preoperative and postoperative outcomes between the two groups. All data were analyzed using SPSS (version 27.0; IBM, Armonk, NY, USA), and statistical significance was set at a *p*-value < 0.05. In this study, 131 and 27 knees were assigned to the mild and severe varus groups, respectively. A 10-point difference in the Knee Society score is clinically important [25] and thus, it would take a 99% statistical power to detect a difference of at least 10 points with a standard deviation of 20 points in the Knee Society Score (α = 0.05).

## 3. Results

**Figure 4:**
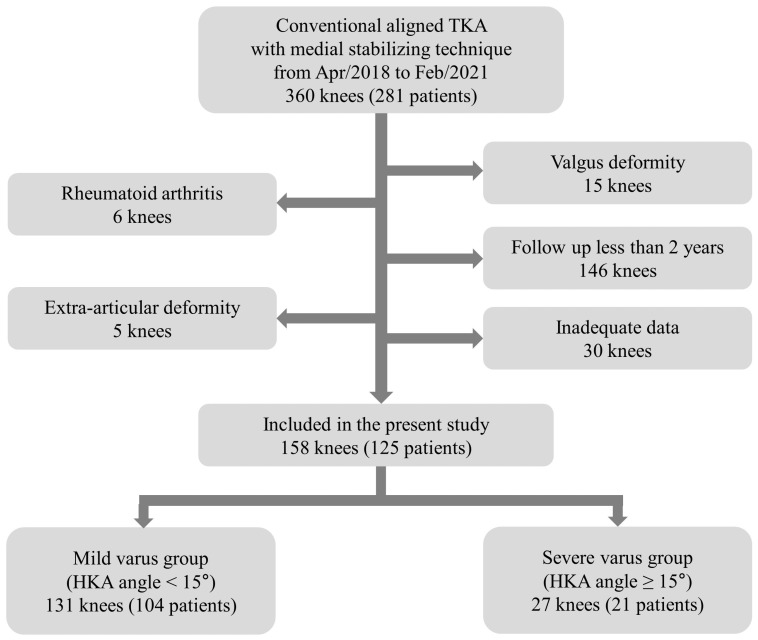
Flow chart describing the patients enrolled in the current study.

Between April 2018 and February 2021, 360 knees (281 patients) were screened. After applying exclusion criteria, 158 knees (125 patients) were enrolled in the current study. Among them, 131 and 27 knees were classified into the mild and severe varus groups, respectively (Figure 4). Demographic and preoperative data are presented in Table 1. All inter- and intraobserver ICCs showed good agreement for the reliability of the radiographic measurements (>0.80). 

**Figure 5:**
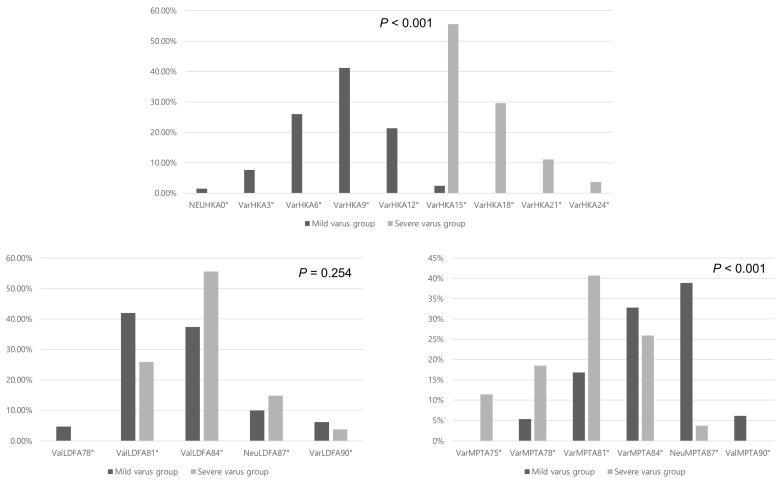
Distribution of the hip-knee-ankle (HKA) angle, lateral distal femoral angle (LDFA), and medial proximal tibial angle (MPTA) phenotype in both groups.

The severe varus group showed a significantly higher varus phenotype with respect to the HKA angle owing to the grouping definition (*p* < 0.001). The proportion of LDFA phenotype was not significantly different between both groups (*p* = 0.254), while the severe varus group showed significantly more varus MPTA phenotype (*p* < 0.001, Figure 5).

With respect to the correlation between the preoperative HKA angle and other preoperative radiographic measurements, a greater preoperative HKA angle was significantly correlated with greater preoperative LDFA (r = 0.219, *p* = 0.006) and lesser preoperative MPTA (r = −0.551, *p* < 0.001). In terms of the correlation between the preoperative HKA angle and postoperative measurements, a greater preoperative HKA angle was associated with a greater postoperative HKA angle (r = 0.239, *p* = 0.003) and femoral component rotation angle (r = −0.271, *p* = 0.001). 

Postoperatively, all clinical outcomes, including ROM, WOMAC, KSKS, and KSFS improved significantly. (Table 2) Only patellar tilt angle was not significantly different between pre- and post-operation. Postoperative HKA angle, LDFA, and MPTA were 1.2° ± 1.7°, 89.8° ± 2.2°, and 89.5° ± 1.8°, respectively. The joint-line distance increased significantly postoperatively (17.2 mm ± 3.8 mm vs. 18.4 mm ± 2.8 mm, *p* < 0.001). Postoperative patellar tilt angle was not significantly correlated with postoperative WOMAC index (r = 0.15, *p* = 0.1), KSKS (r = −0.013, *p* = 0.87), and KSFS (r = 0.065, *p* = 0.42) in all cases. In subgroup analysis, postoperative patellar tilt angle of both mild varus and severe varus groups were not also significantly related to postoperative WOMAC index (mild varus group: r = 0.152, *p* = 0.21; severe varus group: r = 0.054, *p* = 0.788), KSKS index (mild varus group: r = 0.043, *p* = 0.627; severe varus group: r = −0.167, *p* = 0.14), and KSFS index (mild varus group: r = 0.0.067, *p* = 0.447; severe varus group: r = −0.035, *p* = 0.862).

**Figure 6:**
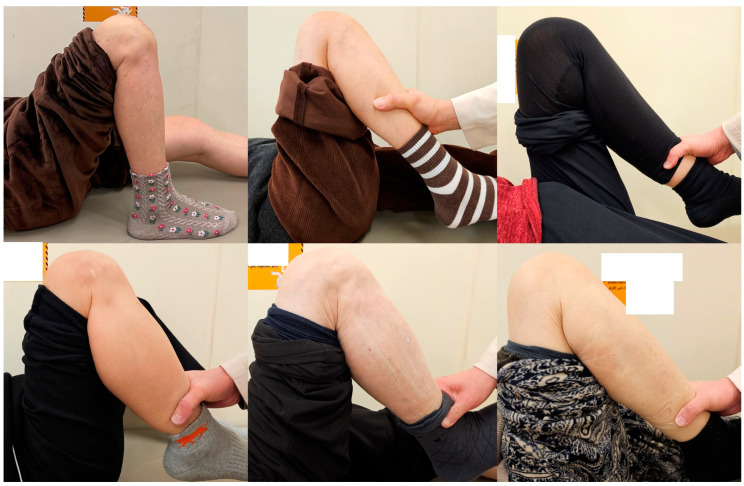
Examples of knee flexion after TKA surgeries.

When comparing the demographic and preoperative data between the two groups, the mean HKA angle and MPTA were significantly different; however, the other parameters were not (Table 3). Postoperatively, the mean HKA angle was significantly greater in the severe varus group; however, the outlier rate of the postoperative HKA angle did not differ. The postoperative extension angle on radiographs was significantly smaller and the femoral component rotation angle was significantly greater in the severe varus group. The joint line distance and the change in joint line distance (from preoperative to postoperative) were similar between the two groups. Postoperative range of motion, WOMAC, KSKS, and KSFS scores did not differ between the groups (Table 4, Figure 6). Aseptic loosening or periprosthetic joint infection was not observed in all cases during follow up period. Revision surgery was also not performed for all patients.

## 4. Discussion

The principal finding of the present study was that the severe varus group showed comparable postoperative clinical and radiographic outcomes, including joint line distance, after MA TKA with MST. 

The strategy of optimal alignment for TKA surgery is still controversial. Conventionally, the concept of MA TKA targeting the alignment of the leg neutrally and implanting a prosthesis parallel to the neutral alignment of the lower leg has been the method of choice [26]. This method yields excellent survival rates and comparable patient satisfaction. However, some patients were dissatisfied with the functional issues. To overcome this issue, new alignment strategies, including kinematic alignment (KA) and restricted KA (rKA), have been introduced [27]. These are aimed at restoring pre-arthritic alignment, which might lead to more natural knee kinematics. Therefore, the functional outcomes of the patients can be improved [28]. It is important to understand knee phenotypes in personalized TKA approaches. Several studies have reported that knee phenotypes in patients undergoing TKA are heterogeneous; therefore, a single alignment strategy is not sufficient for all patients [19]. According to previous studies, we believe understanding individual knee phenotypes is necessary to improve postoperative results. In our study, the MPTA in the severe varus group was significantly lower than that in the mild varus group, whereas the LDFA was not statistically different. The distribution of the MPTA phenotype was also significantly different between the two groups. Our results indicated that the tibial vara rather than the femoral vara was correlated with lower extremity varus deformity in the female Asian population.

Conventionally, achieving rectangular soft tissue balancing is important for successful MA TKA. Medial soft tissue release is usually performed to achieve a rectangular soft tissue balance in TKA for varus knee deformities [2]. Excessive medial side release is often necessary, depending on the medial side tightness. Extensive medial soft tissue release can induce midflexion instability or joint line elevation, both of which are associated with poor postoperative patients’ functional outcomes [4,29]. Joint line elevation occurs when the tibiofemoral joint line rises without a change in the length of the patellar tendon due to the use of a thick polyethylene insert due to excessive soft tissue release [30]. This phenomenon is associated with decreased postoperative ROM and poor functional outcomes [31]. The main concept of TKA with MST is to minimize medial soft tissue release and allow residual lateral side laxity to avoid the potential risk of joint line elevation and associated poor outcomes [5]. Numerous studies have reported that TKA using this technique has excellent postoperative outcomes without joint line elevation or subjective instability. Tanaka et al. investigated the effect of the varus-valgus gap angle and laxity in extension and flexion on postoperative clinical outcomes after 100 cases of TKA without any medial side release [32]. The average varus-valgus angles had a residual imbalance of 1.3° varus and 2.8° varus in flexion and extension, respectively. The varus-valgus joint gap angle and knee laxity was not influenced on postoperative ROM and Knee Society Scores. Ishibashi et al. compared between TKA with MST and gap-balancing technique [5]. MST group showed significantly less medial extension gap. Moreover, the gap difference between medial and lateral extension was significantly greater in the MST group. The joint line changes and insert thickness were significantly less in the MST group. In terms of clinical outcomes, MST group showed better postoperative ROM than gap-balancing group. Thy concluded the MST could avoid knee joint line change and might increase postoperative ROM. Tsubosaka et al. had compared medial preserving gap technique and conventional measured resection technique in terms of postoperative knee stability using stress radiographs and functional outcomes [6]. Medial preserving gap technique showed better postoperative mediolateral stability and functional outcomes. Nagai et al. had also compared the mediolateral gap change between TKA with MST and conventional measured resection technique [33]. They found TKA with MST showed more consistent medial and lateral joint gap during mid-to-deep knee flexion (30°–90° in medial, 30°–120° in lateral) than conventional measured resection technique. Despite of numerous studies of TKA with MST, previous studies have rarely performed subgroup analysis for the results of MST TKA in severe varus deformity. In our study, the severe varus group showed comparable postoperative clinical and radiographic outcomes, including joint line distance, after MA TKA with MST. Our results suggest that TKA using the MST can be safely performed regardless of the severity of the varus deformity. 

A previous study demonstrated that the severity of the preoperative varus alignment was closely related to the mediolateral gap difference [11]. One major concern in allowing for lateral laxity is the possibility of hyperextension after TKA. Excessive residual posterolateral ligament laxity may induce postoperative recurvatum deformities [34]. Postoperative extension angle on radiographs was significantly lesser in the severe varus group in our study (mild varus group vs. severe varus group, 3.2° ± 2.3° vs. 2.0° ± 2.9°, *p* = 0.02), however, the mean postoperative extension angles of both group were mild flexion contracture rather than hyperextension. Sekiya et al. demonstrated that the residual lateral side laxity during TKA surgery in patients with varus deformities spontaneously diminished after TKA with neutral alignment [35]. We believe that it is difficult to determine whether the residual lateral laxity is induced by postoperative hyperextension. A large-scale investigation is needed to clarify the effect of the mediolateral gap balance more precisely on the extension angle.

The gap technique with rectangular gap balancing is potentially associated with joint line elevation or an internally rotated femoral component, which is related to anterior knee pain [36]. These disadvantages are associated with the release of medial soft tissue to obtain a rectangular gap balance. However, the gap technique using MST is not related to these potential problems. In our study, the severe varus group showed 0.7° greater external rotation than the mild varus group, which was statistically significant. However, the difference was less than 1°. A previous study reported that the rotational alignment of the femoral component was not a significant factor in patient clinical outcomes [37]. We believe that a 0.7° difference in the rotation angle of the femoral component does not significantly affect patient outcomes. We believe that the gap technique can prevent excessive internal rotation of the femoral component and joint line elevation with MST TKA.

Our study has a few limitations. First, although it was not our intention, only female patients were enrolled. Twenty-one male patients were performed the TKA with MST, however, all of them were lost to follow-up. Therefore, it is difficult to determine whether our results apply to male patients. Second, numerous studies have reported excellent postoperative outcomes following MST TKA using several surgical methods. However, its longevity has not been thoroughly investigated. Our results revealed excellent postoperative results in patients with preoperative severe varus deformity, except for the implant survival rate, due to the relatively short follow-up period. A longer follow-up study is needed to interpret implant longevity after MST TKA in patients with severely varus knees. Third, our study demonstrated that the major bony deformity in severe varus knees was due to the proximal tibial phenotype rather than that of the distal femur. Only Asian populations were included in the current study. Ethnic differences could exist with respect to phenotypes in severe varus knees; therefore, investigations in different races are required. Fourth, most of the patients enrolled in the current study had to visit the hospital for follow-up during the coronavirus disease 2019 (COVID-19) pandemic. Few patients were lost to follow-up. Among the 360 knees initially screened, 146 (41%) were excluded owing to loss to follow-up. Therefore, selection bias could exist. Despite several limitations, our study demonstrated that MA TKA with MST for severely varus knees showed comparable clinical and radiographic outcomes, without significant joint line elevation, to those for mildly varus knees.

## 5. Conclusions

The severe varus group showed clinical and radiographic outcomes comparable to those of the mild varus group after MA TKA with MST.

## Figures and Tables

**Table 1:** Demographic and preoperative data.

Age, year	71.1 ± 5.2 (57–82)
Sex, male:female	0:158
Body mass index, kg/m^2^	26.7 ± 3.1 (20–37.5)
Direction, right:left	80:78
Mean follow up period, months	29.6 ± 8.8 (23.7–56.7)
Preoperative HKA angle, °	9.9 ± 4.3 (0.6–23)
Preoperative LDFA, °	83.4 ± 2.5 (78.5–89.9)
Preoperative MPTA, °	84.0 ± 3.3 (73–90)
Preoperative tibial slope, °	9.1 ± 3.0 (1–16.2)
Preoperative patellar tilt angle, °	9.8 ± 5.1 (−0.9–22.3)

HKA, hip-knee-ankle; LDFA, lateral distal femoral angle; MPTA, medial proximal tibial angle.

**Table 2:** Comparison of the clinical and radiographic outcomes between pre- and post-operation.

	Preoperation	Postoperation	*p* Value
Range of motion, °	132.6 ± 21.3	142.3 ± 13.3	<0.001
WOMAC index	55.3 ± 16.6	8.3 ± 4.9	<0.001
KSKS	58.8 ± 5.6	93.9 ± 7.1	<0.001
KSFS	51.7 ± 7.4	81.7 ± 15.6	<0.001
HKA angle, °	9.9 ± 4.3	1.2 ± 1.7	<0.001
LDFA, °	83.4 ± 2.5	89.8 ± 2.2	<0.001
MPTA, °	84.0 ± 3.3	89.5 ± 1.8	<0.001
Tibial slope, °	9.1 ± 3.0	4.0 ± 2.2	<0.001
Patellar tilt angle, °	9.8 ± 5.1	9.2 ± 4.6	0.07
Extension angle on full extension lateral radiographs, °	4.4 ± 2.9	3.0 ± 2.4	<0.001
Femoral condylar offset, mm	28.8 ± 3.4	31.3 ± 3.0	<0.001
Joint line distance, mm	17.2 ± 3.8	18.4 ± 2.8	<0.001

WOMAC, Western Ontario and McMaster Universities Osteoarthritis; KSKS, Knee Society Knee Score; KSFS, Knee Society Function Score; HKA, hip-knee-ankle; LDFA, lateral distal femoral angle; MPTA, medial proximal tibial angle.

**Table 3:** Comparison of preoperative outcomes between mild and severe varus groups.

	Mild Varus Group	Severe Varus Group	*p* Value
Number of knees	131	27	
Age, year	71.3 ± 5.3	70.0 ± 4.9	0.215
Body mass index, kg/m^2^	26.6 ± 3.2	26.9 ± 2.7	0.632
Direction, right:left	68:63	12:15	0.53
Range of motion, °	133.2 ± 20.8	129.6 ± 24.0	0.429
WOMAC index	55.9 ± 17.5	53.9 ± 11.2	0.637
KSKS	59.1 ± 5.7	57.1 ± 5.1	0.102
KSFS	50.7 ± 7.3	50.4 ± 6.6	0.786
HKA angle, °	8.4 ± 2.9	16.9 ± 2.4	<0.001
LDFA, °	83.3 ± 2.6	83.9 ± 1.9	0.225
MPTA, °	84.7 ± 2.8	80.7 ± 3.2	<0.001
Tibial slope, °	9.1 ± 2.9	9.0 ± 3.3	0.897
Patellar tilt angle, °	9.8 ± 5.1	9.8 ± 5.5	0.997
Extension angle on full extension lateral radiographs, °	4.5 ± 3.0	4.0 ± 2.6	0.384
Femoral condylar offset, mm	28.7 ± 3.4	29.4 ± 3.1	0.304
Joint line distance, mm	17.2 ± 3.8	17.2 ± 3.7	0.99

WOMAC, Western Ontario and McMaster Universities Osteoarthritis; KSKS, Knee Society Knee Score; KSFS, Knee Society Function Score; HKA, hip-knee-ankle; LDFA, lateral distal femoral angle; MPTA, medial proximal tibial angle.

**Table 4:** Comparison of postoperative outcomes between mild and severe varus groups.

	Mild Varus Group	Severe Varus Group	*p* Value
Range of motion, °	142.2 ± 13.8	142.6 ± 10.6	0.882
WOMAC index	8.6 ± 5.0	7.1 ± 4.1	0.181
KSKS	94.2 ± 7.0	92.3 ± 7.2	0.133
KSFS	81.3 ± 16.0	83.9 ± 13.5	0.215
HKA angle, °	1.0 ± 1.4	2.2 ± 2.6	0.001
Outlier of HKA angle, % (n)	8.4% (11)	14.8% (4)	0.47
LDFA, °	89.6 ± 2.1	90.4 ± 1.8	0.088
Outlier of LDFA angle, % (n)	6.9% (9)	7.4% (2)	0.92
MPTA, °	89.6 ± 1.6	89.2 ± 1.5	0.232
Outlier of MPTA angle, % (n)	4.6% (6)	3.7% (1)	0.84
Tibial slope, °	3.9 ± 2.2	4.3 ± 2.3	0.419
Patellar tilt angle, °	9.0 ± 4.7	10.1 ± 4.0	0.263
Extension angle on full extension lateral radiographs, °	3.2 ± 2.3	2.0 ± 2.9	0.02
Femoral condylar offset, mm	31.2 ± 3.0	32.0 ± 2.6	0.142
Joint line distance, mm	18.4 ± 2.8	18.6 ± 2.7	0.676
Change of joint line distance, mm	1.1 ± 4.0	1.4 ± 3.4	0.756
Femoral component rotation angle, °	−1.7 ± 1.0	−1.0 ± 1.3	0.018

WOMAC, Western Ontario and McMaster Universities Osteoarthritis; KSKS, Knee Society Knee Score; KSFS, Knee Society Function Score; HKA, hip-knee-ankle; LDFA, lateral distal femoral angle; MPTA, medial proximal tibial angle.

## Data Availability

The data presented in this study are available on request from the corresponding author. The data are not publicly available due to policy of our institution.
